# Supplementary figures and images for: Youthful systemic milieu in younger recipients alleviates acute kidney injury via attenuating apoptosis and oxidative stress in a rat kidney transplantation model
Source: PLoS One. 2025 Sep 23;20(9):e0331020. doi: 10.1371/journal.pone.0331020 (PMC12456805; doi:10.1371/journal.pone.0331020)

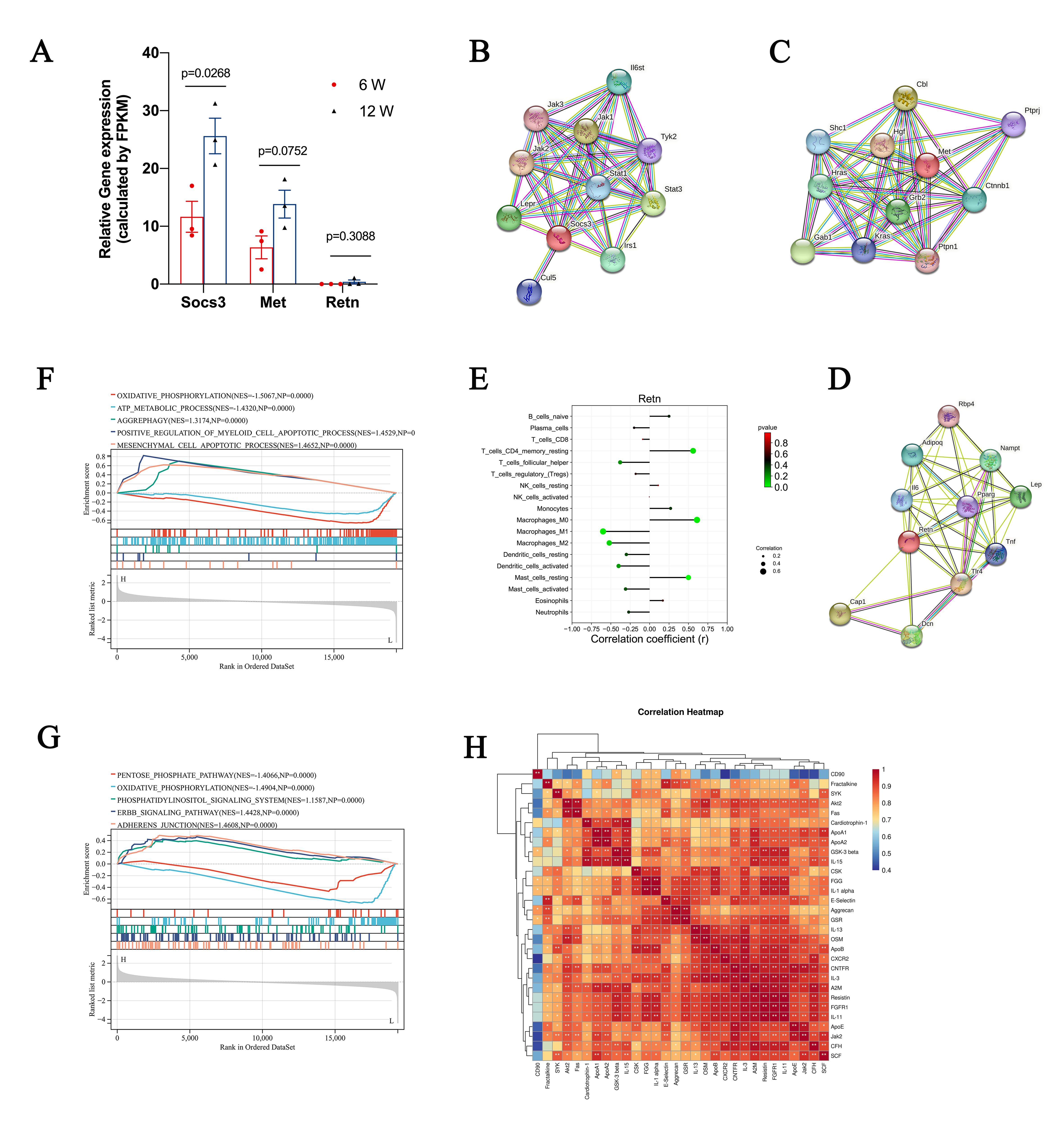

Supplement: S1 Fig — Protein-protein interaction (PPI) network of Socs3 (B), Met (C), and Retn (D) from STRING database. (E) Correlation analysis of Retn and 22 types of immune cells. Single gene GSEA analyses of biological processes enrichments (F) and KEGG pathways (G) based on high- (≥ 50%) and low-expressed (< 50%) Retn. (H) Correlation heatmap of top 30 identified cytokines. (TIF) [file pone.0331020.s001.tif]
