# Supplementary material for: Youthful systemic milieu in younger recipients alleviates acute kidney injury via attenuating apoptosis and oxidative stress in a rat kidney transplantation model
Source: PLoS One. 2025 Sep 23;20(9):e0331020. doi: 10.1371/journal.pone.0331020 (PMC12456805; doi:10.1371/journal.pone.0331020)
Supplement: S1 Table — (DOCX) [file pone.0331020.s002.docx]

**Supplementary table 1** the primers of rat Akt2, ApoA1, and ApoE

|  | Forward (5'-3') | Reverse (5'-3') |
| --- | --- | --- |
| GAPDH | CTGGAGAAACCTGCCAAGTATG | GGTGGAAGAATGGGAGTTGCT |
| Akt2 | CCTTATGCTGGACAAAGATGGC | CCGTAGTCATTGTCCTCTAGCAC |
| ApoA1 | AACAGCTAGGCCCAGTGACTCA | TCCTCGGCCACAACCTTTAGAT |
| ApoE | TGACGGTACTGATGGAGGACACT | CCAGCATGGTGTTTACCTCGTT |
