# Supplementary material for: Youthful systemic milieu in younger recipients alleviates acute kidney injury via attenuating apoptosis and oxidative stress in a rat kidney transplantation model
Source: PLoS One. 2025 Sep 23;20(9):e0331020. doi: 10.1371/journal.pone.0331020 (PMC12456805; doi:10.1371/journal.pone.0331020)
Supplement: S3 File — (DOCX) [file pone.0331020.s005.docx]

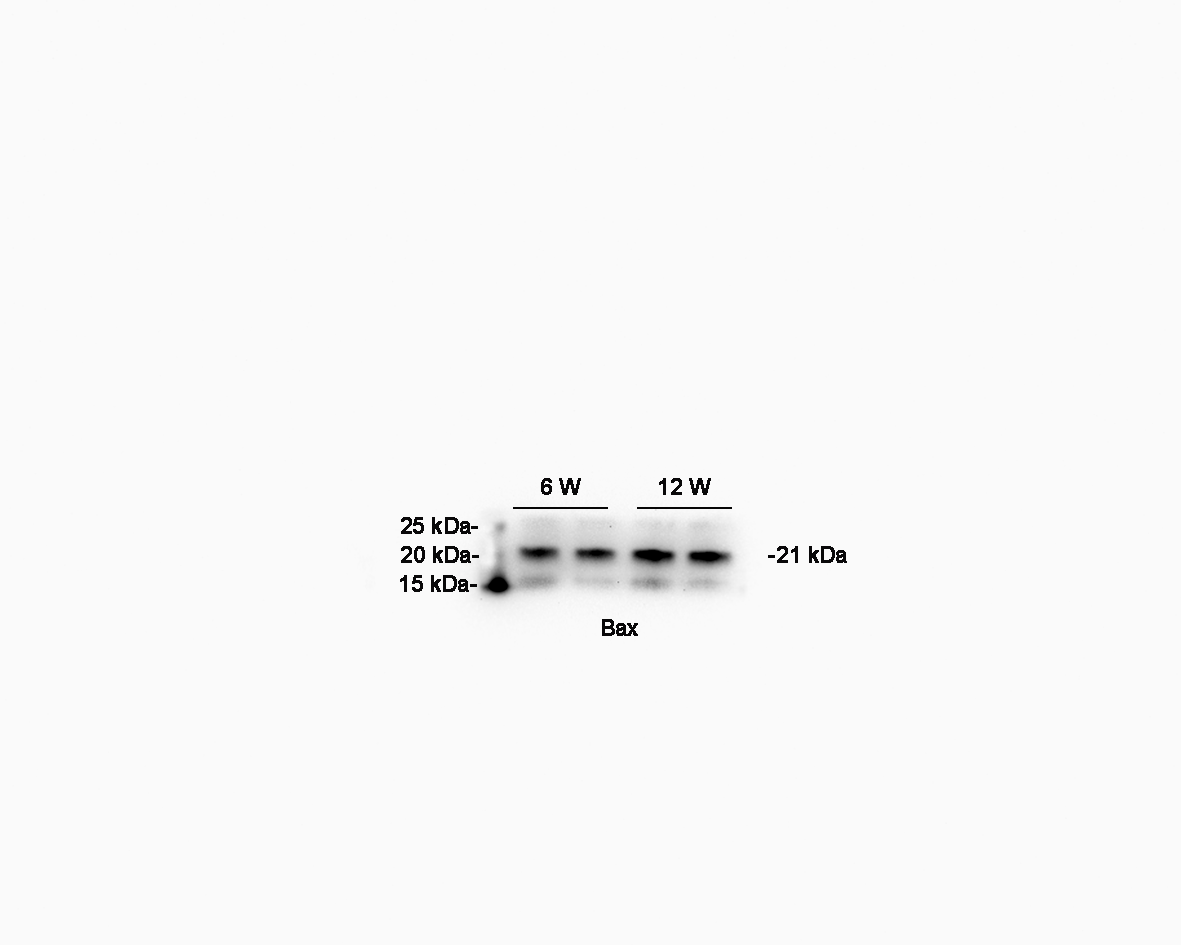


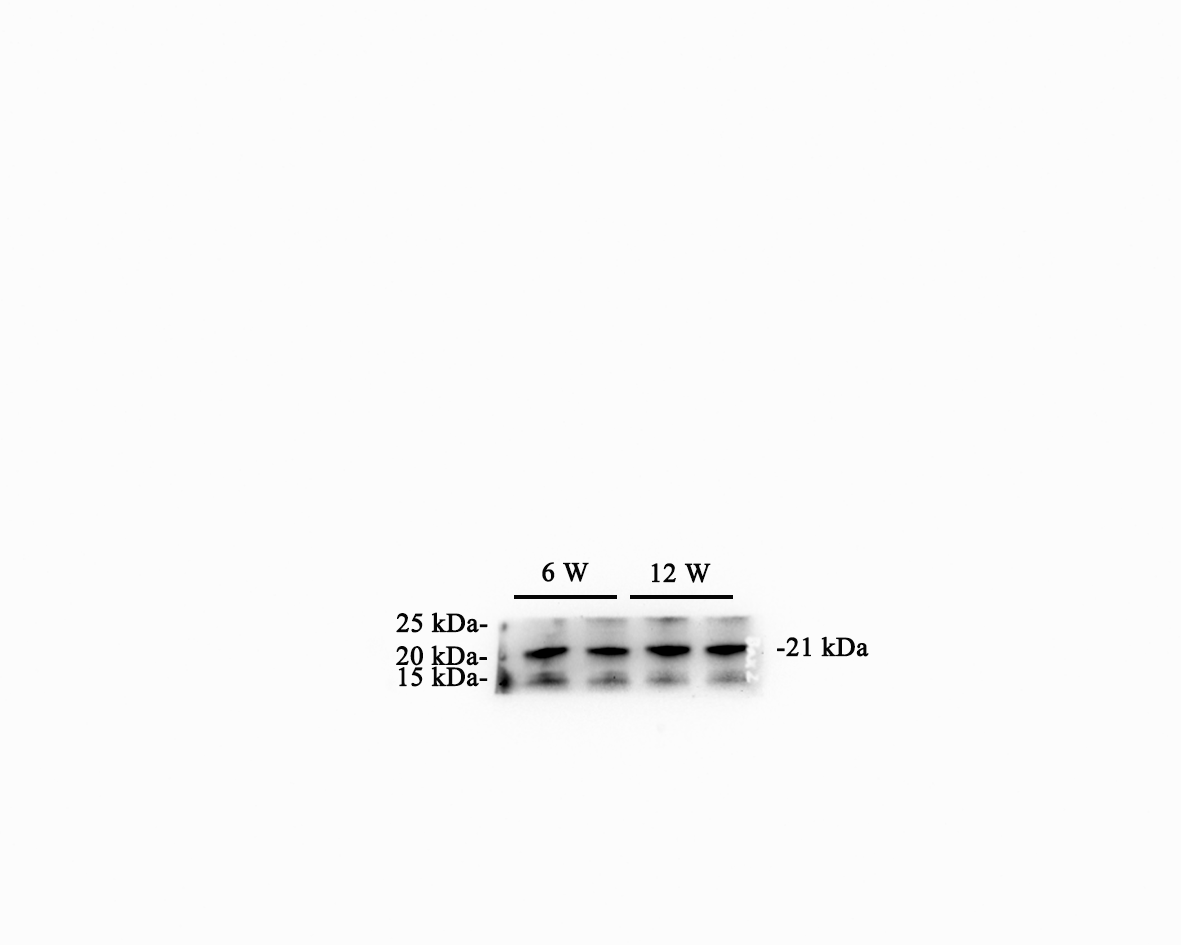


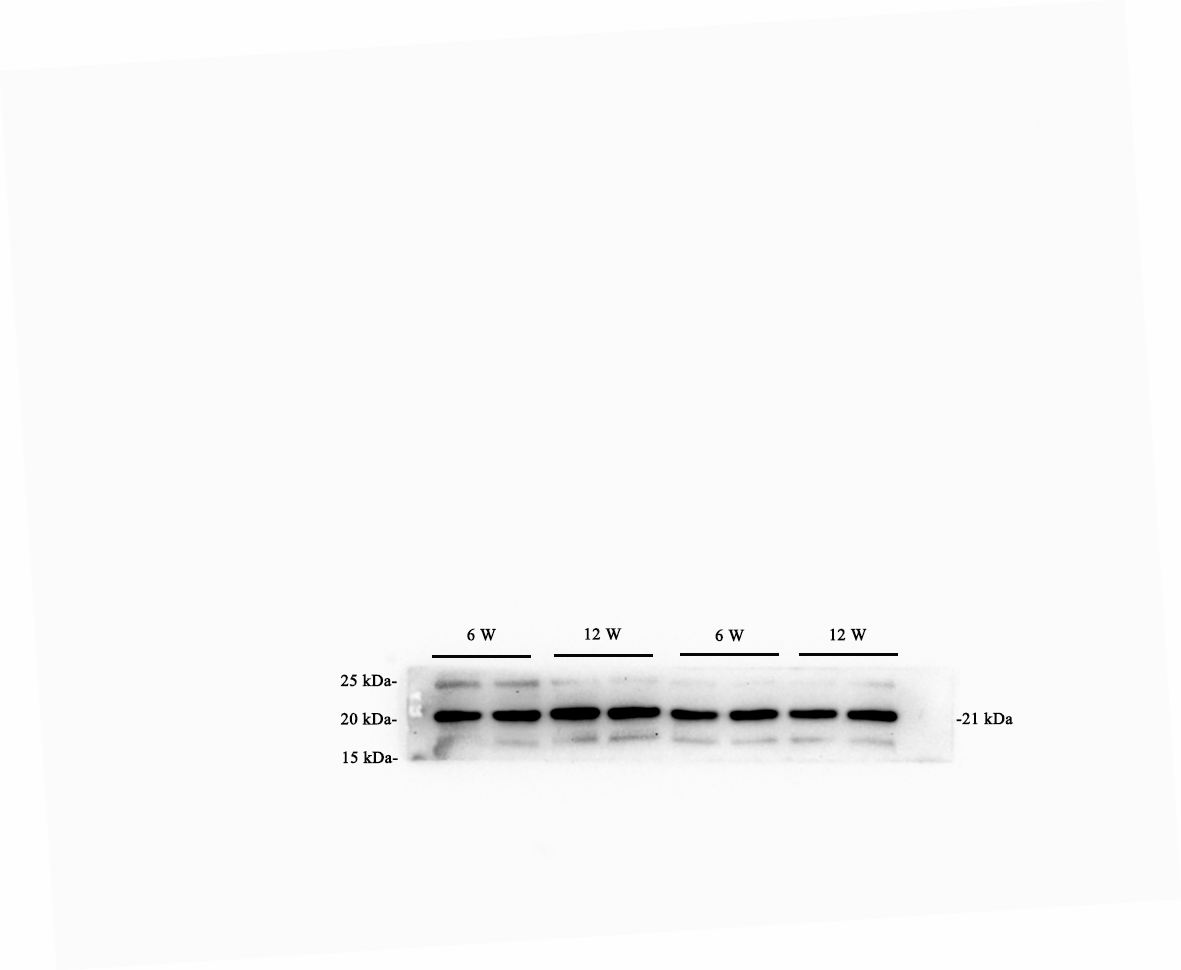


Figure 3G-Bax


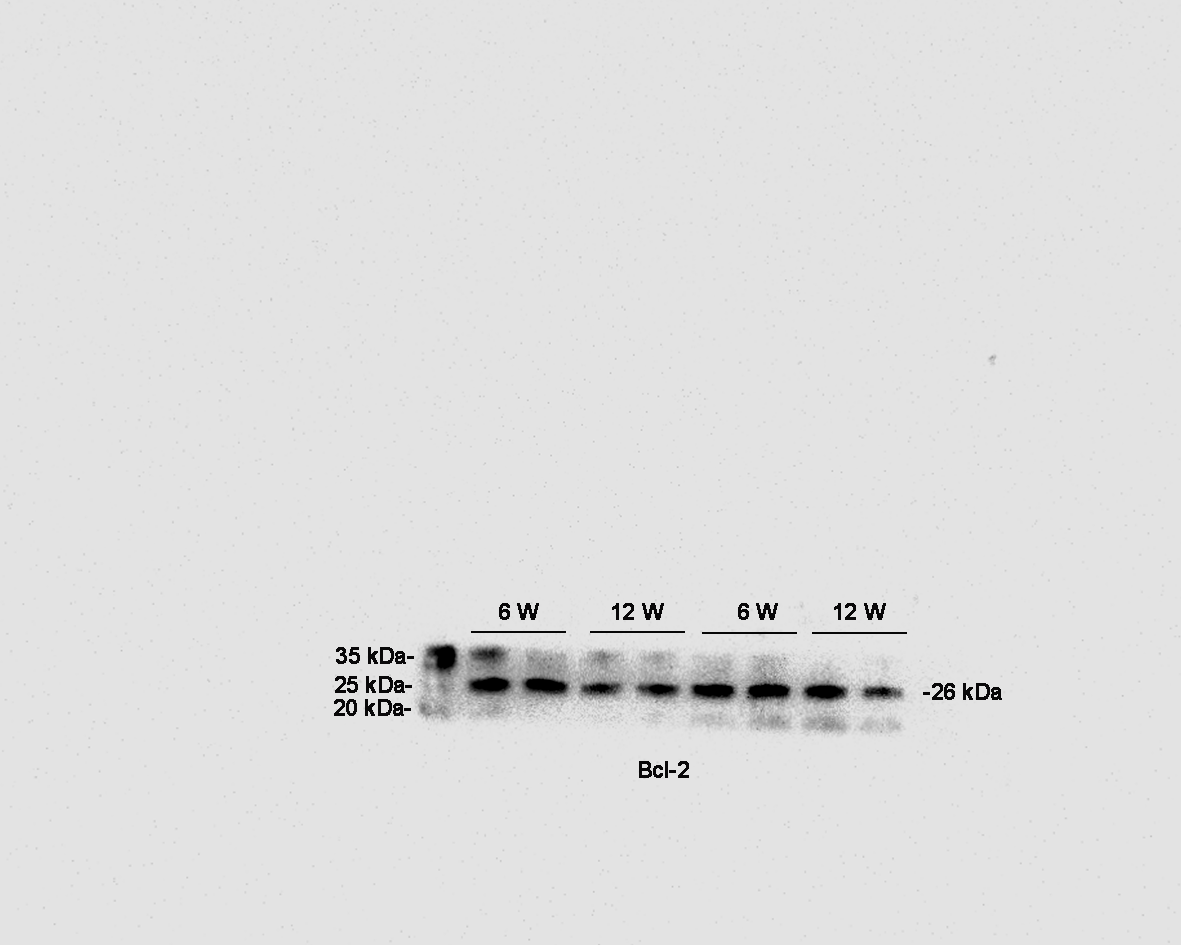


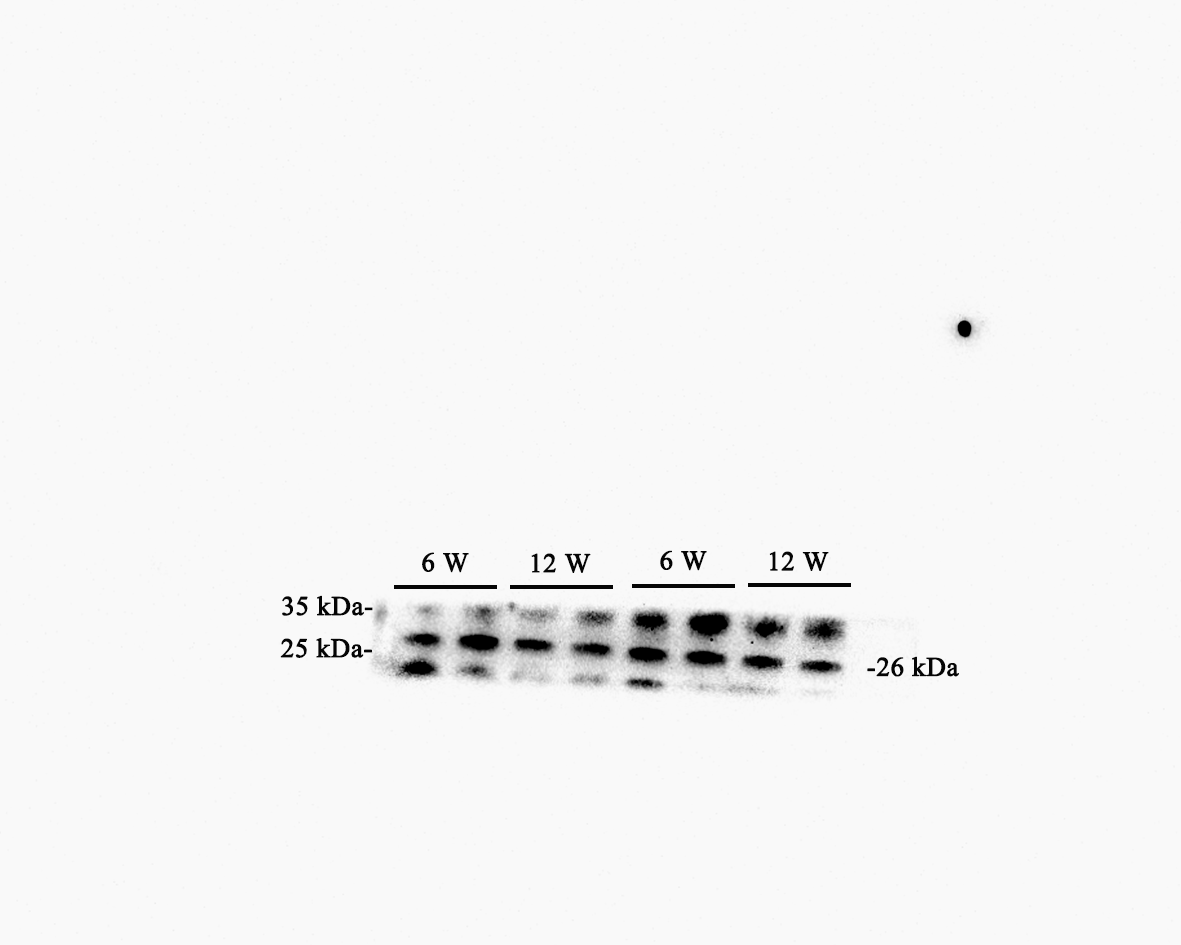


Figure 3G-Bcl-2


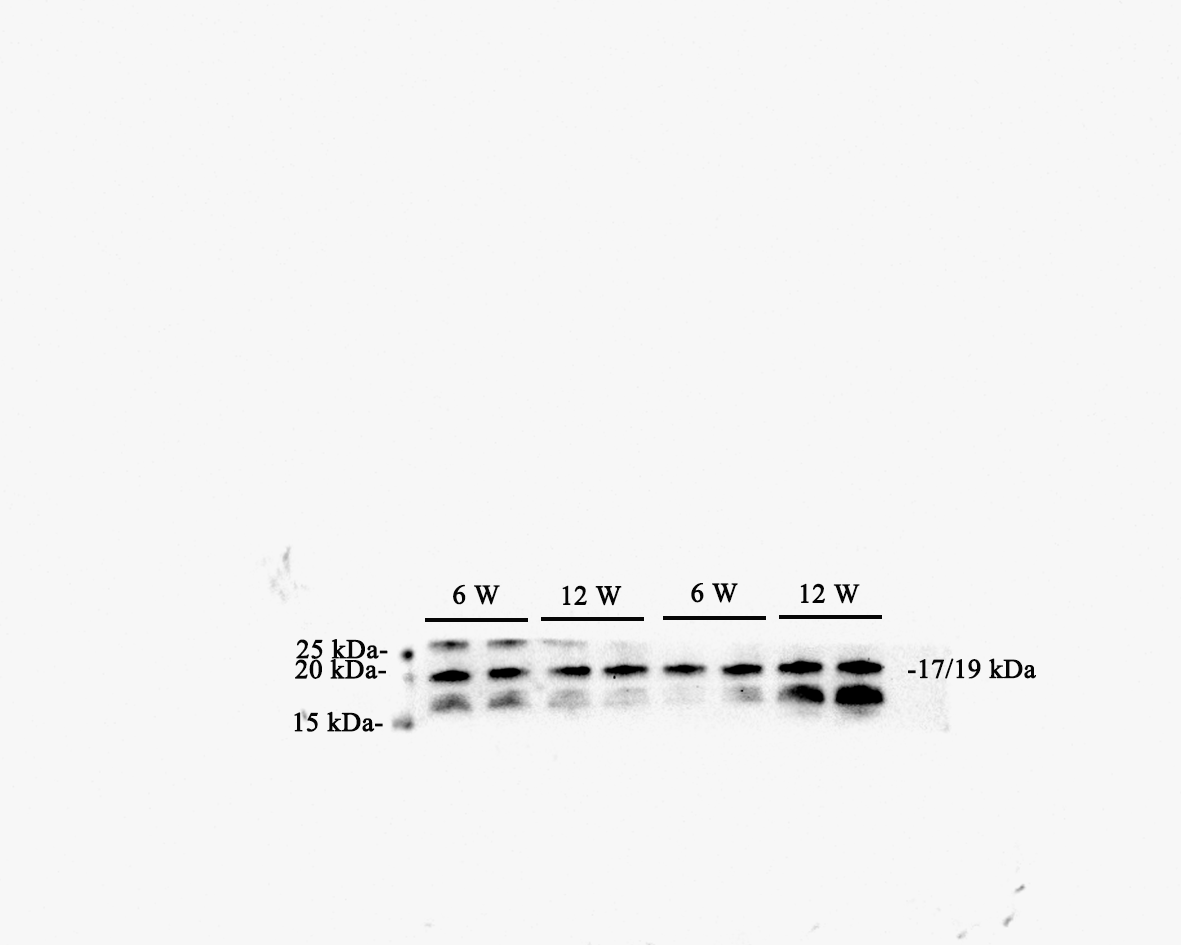


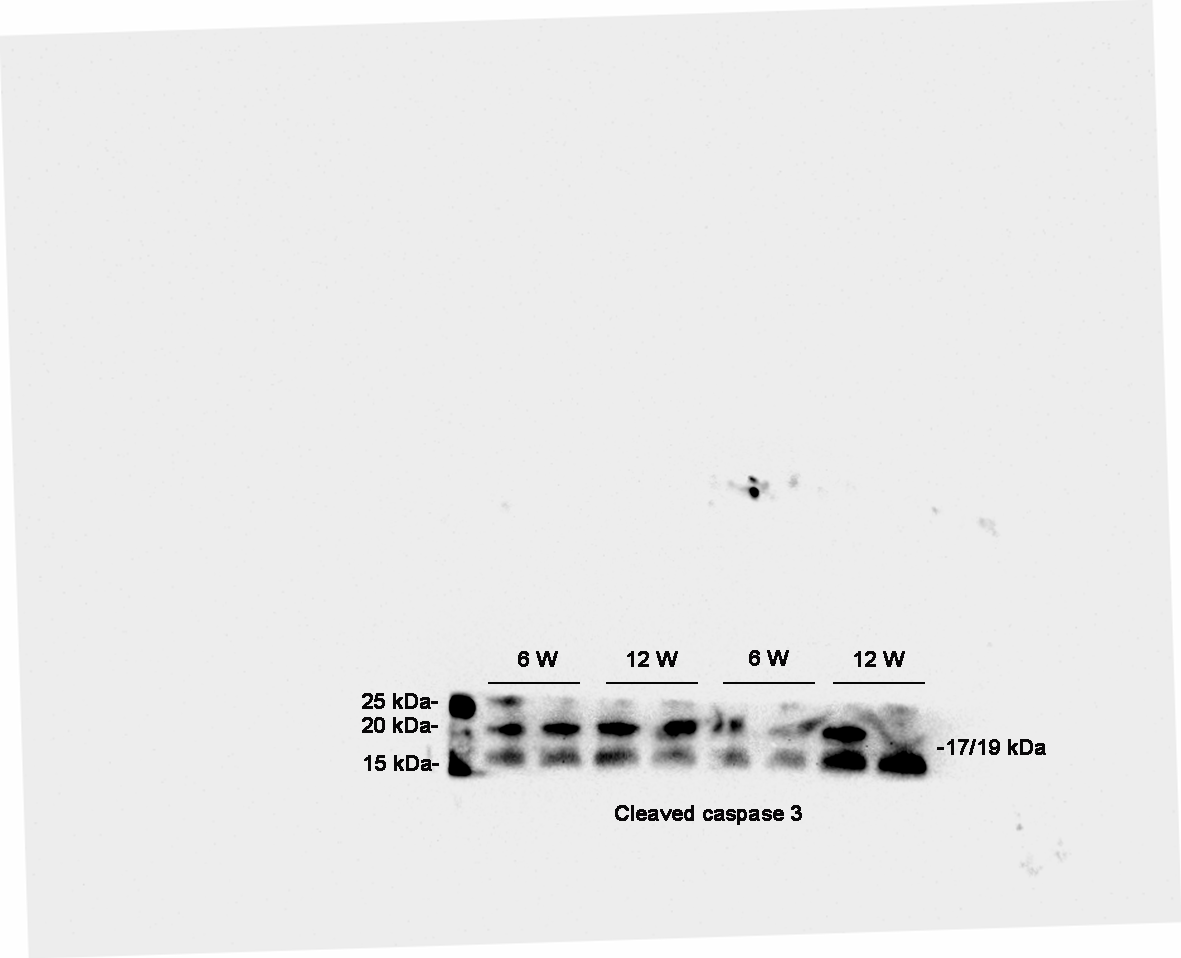


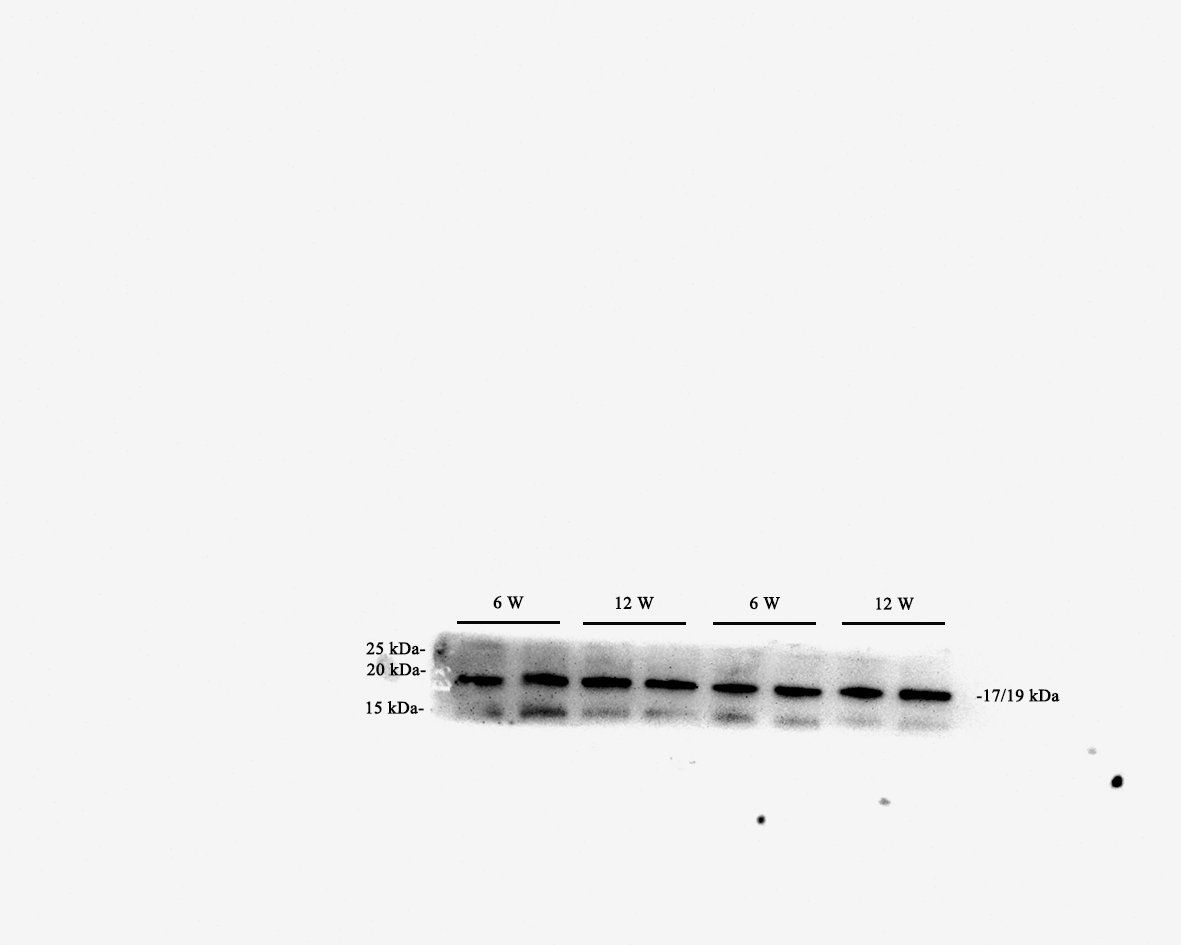


Figure 3G-cleaved caspase 3


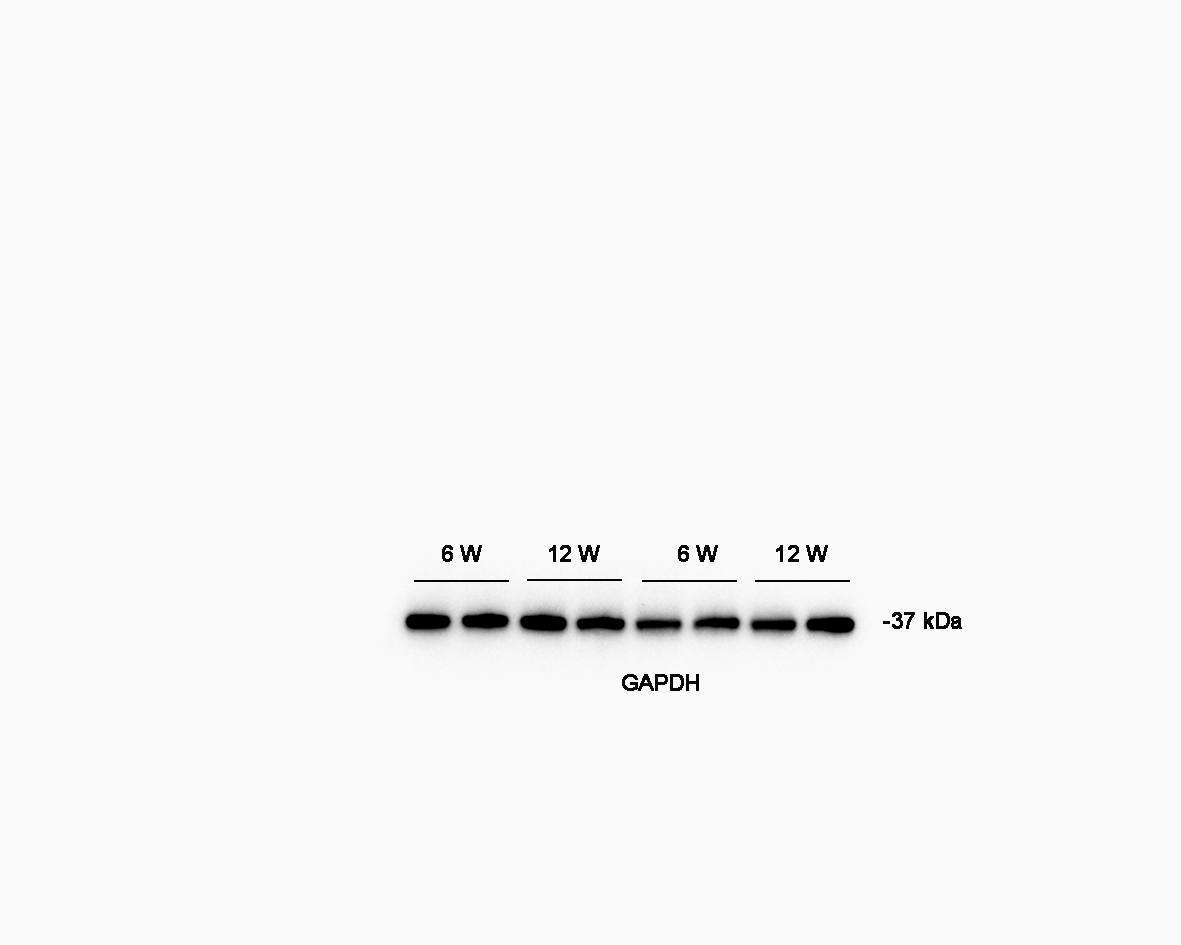


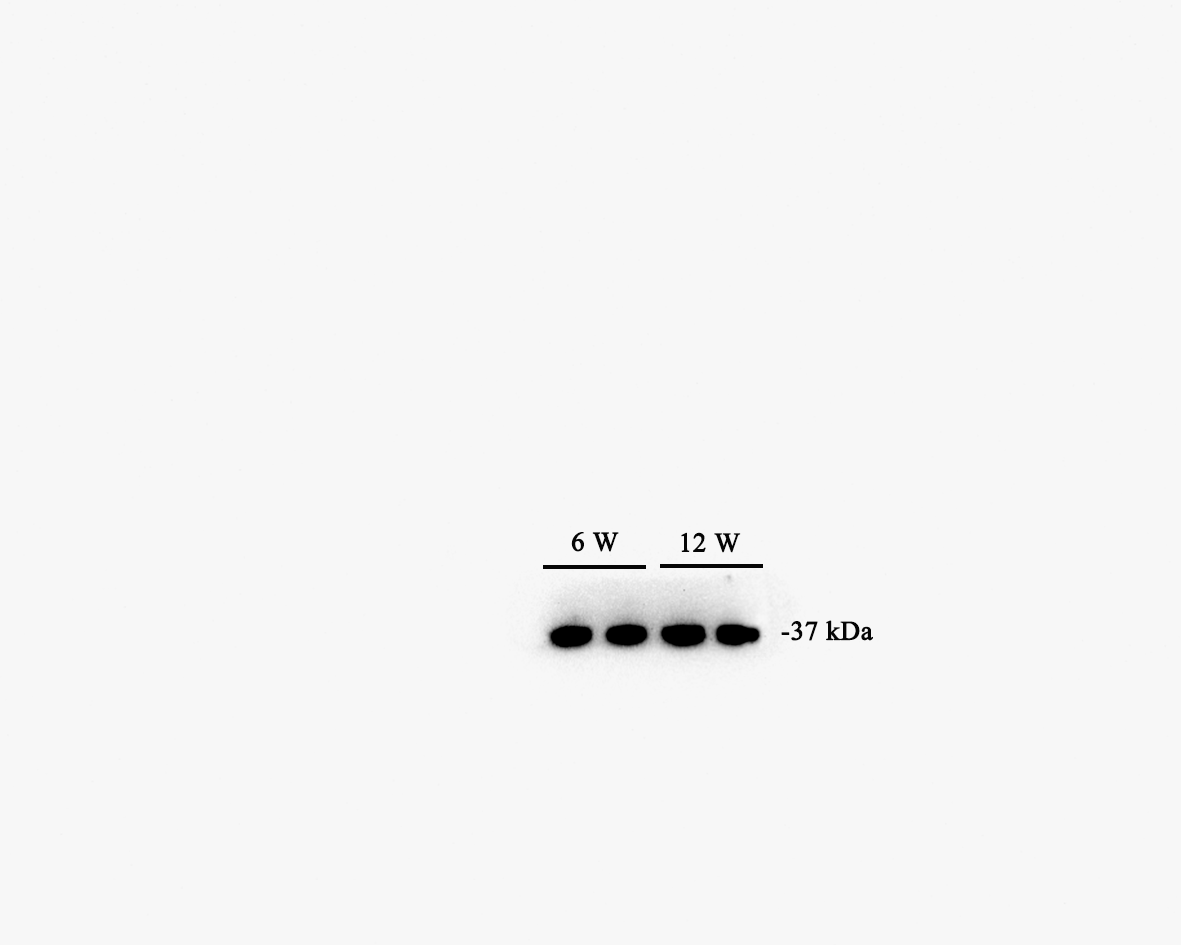


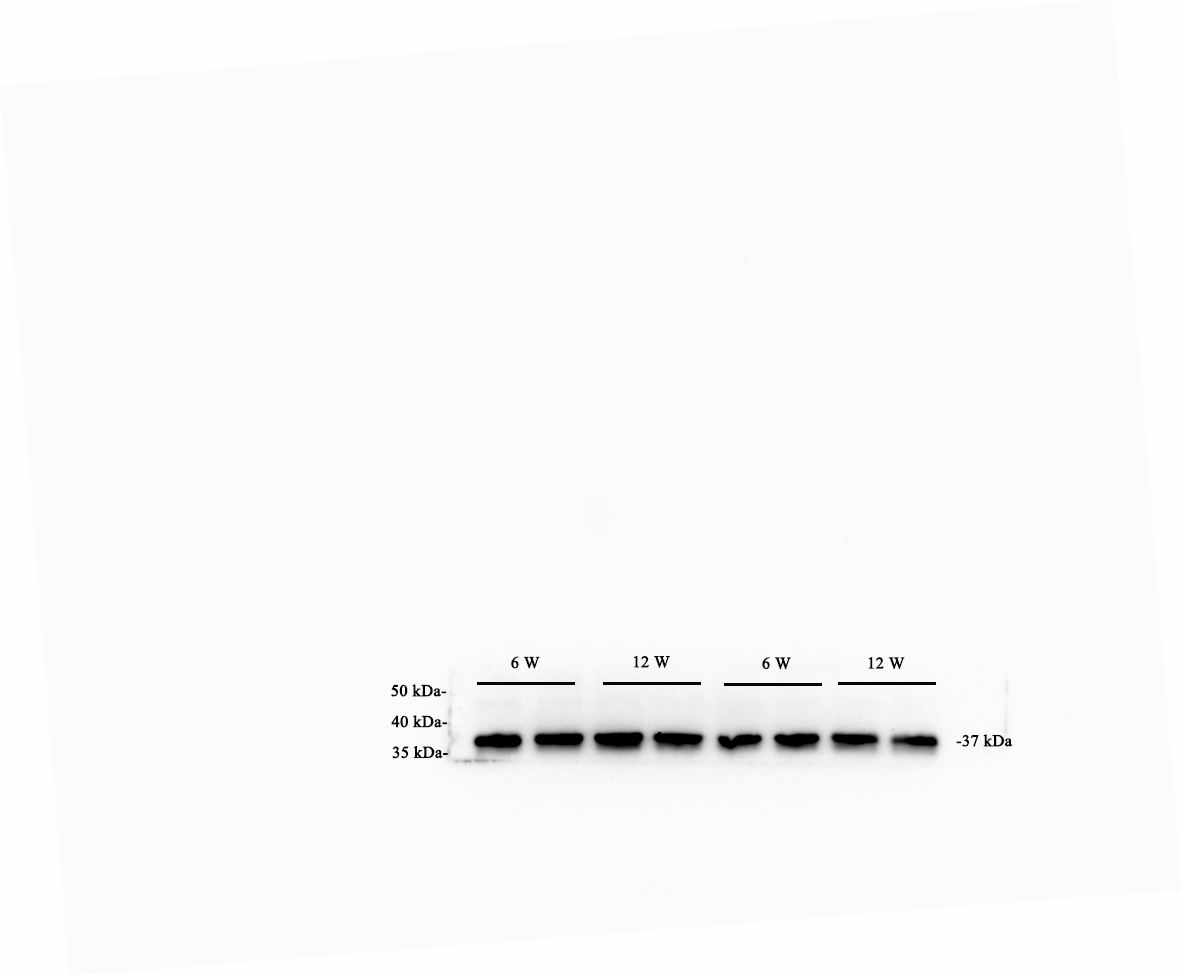


Figure 3G-GAPDH


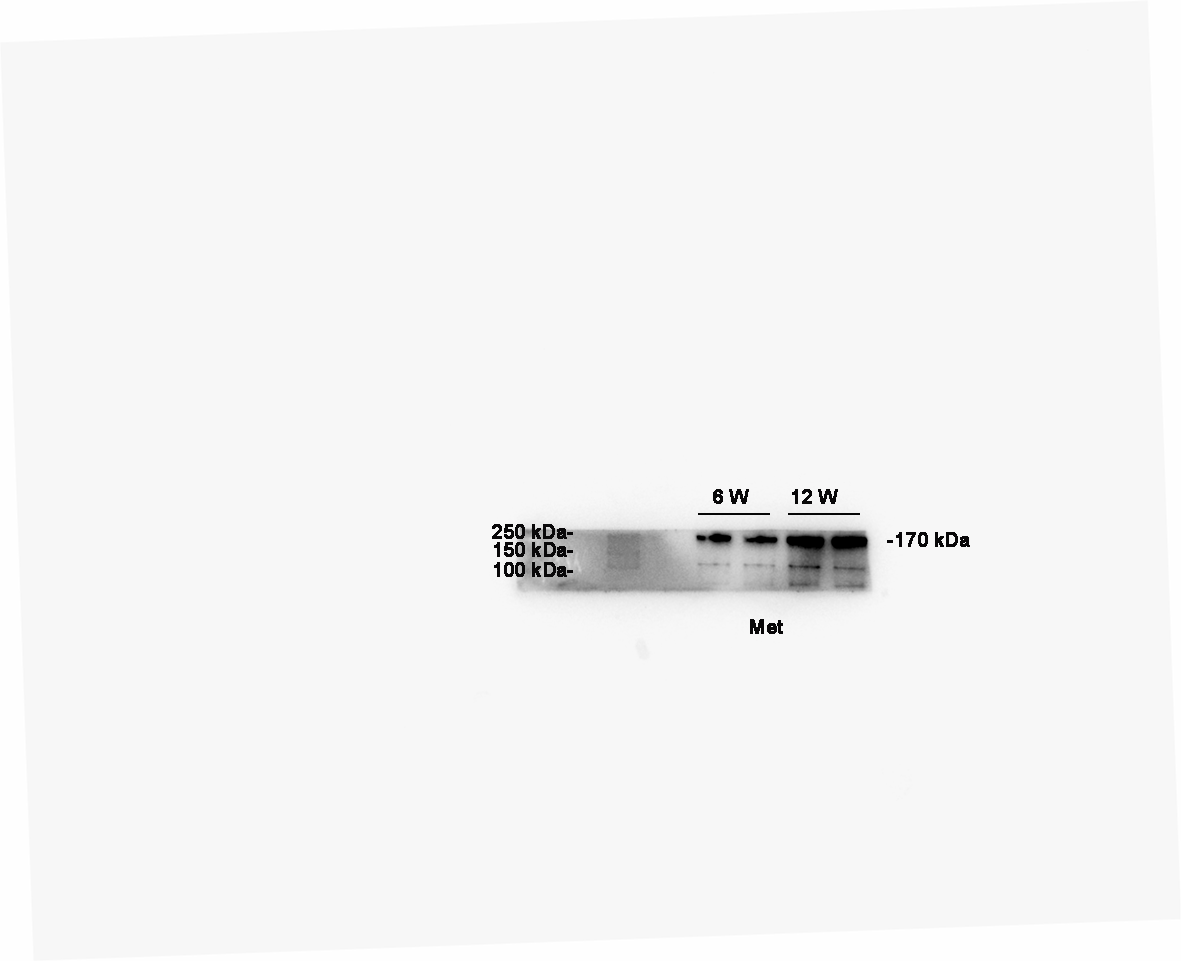


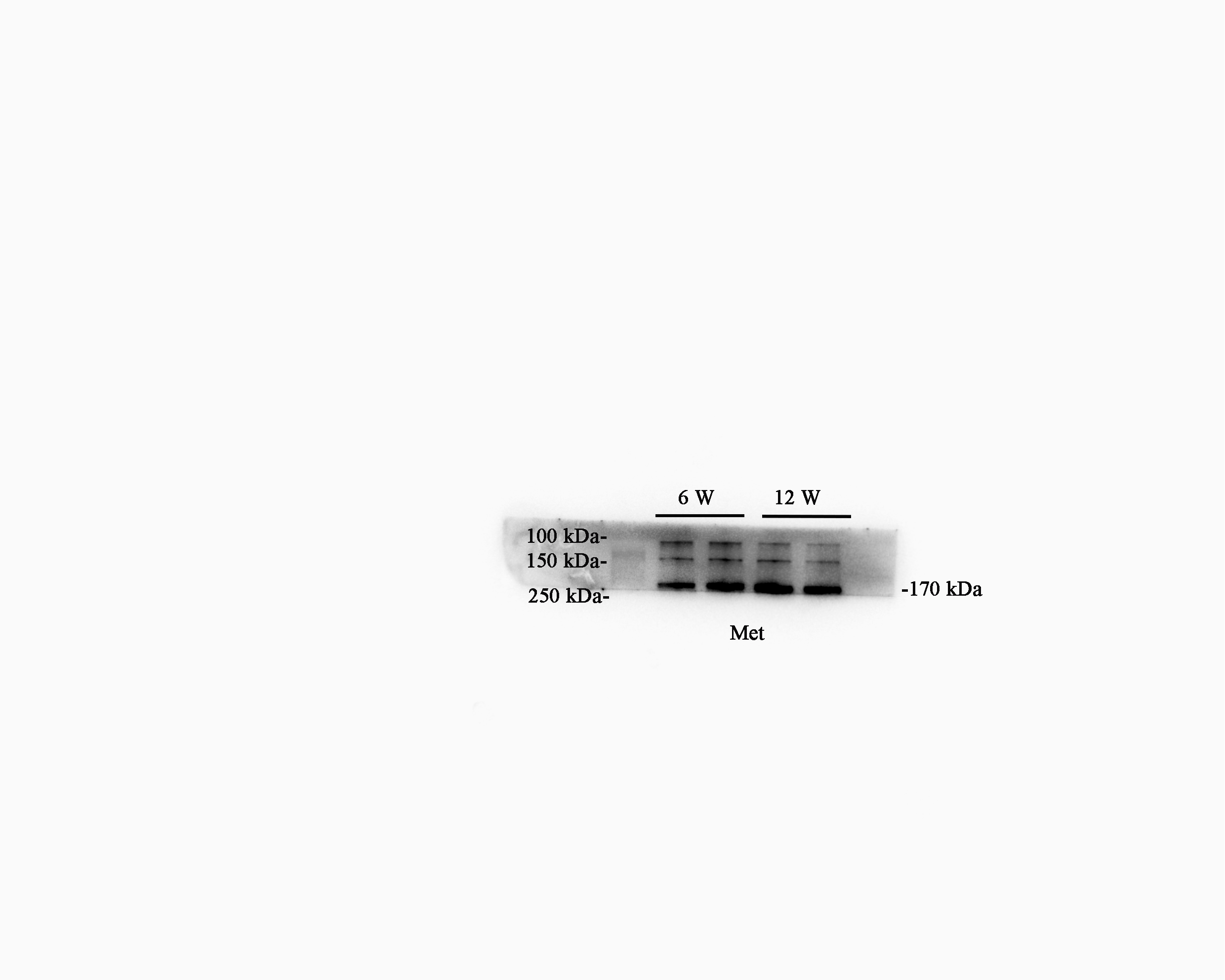


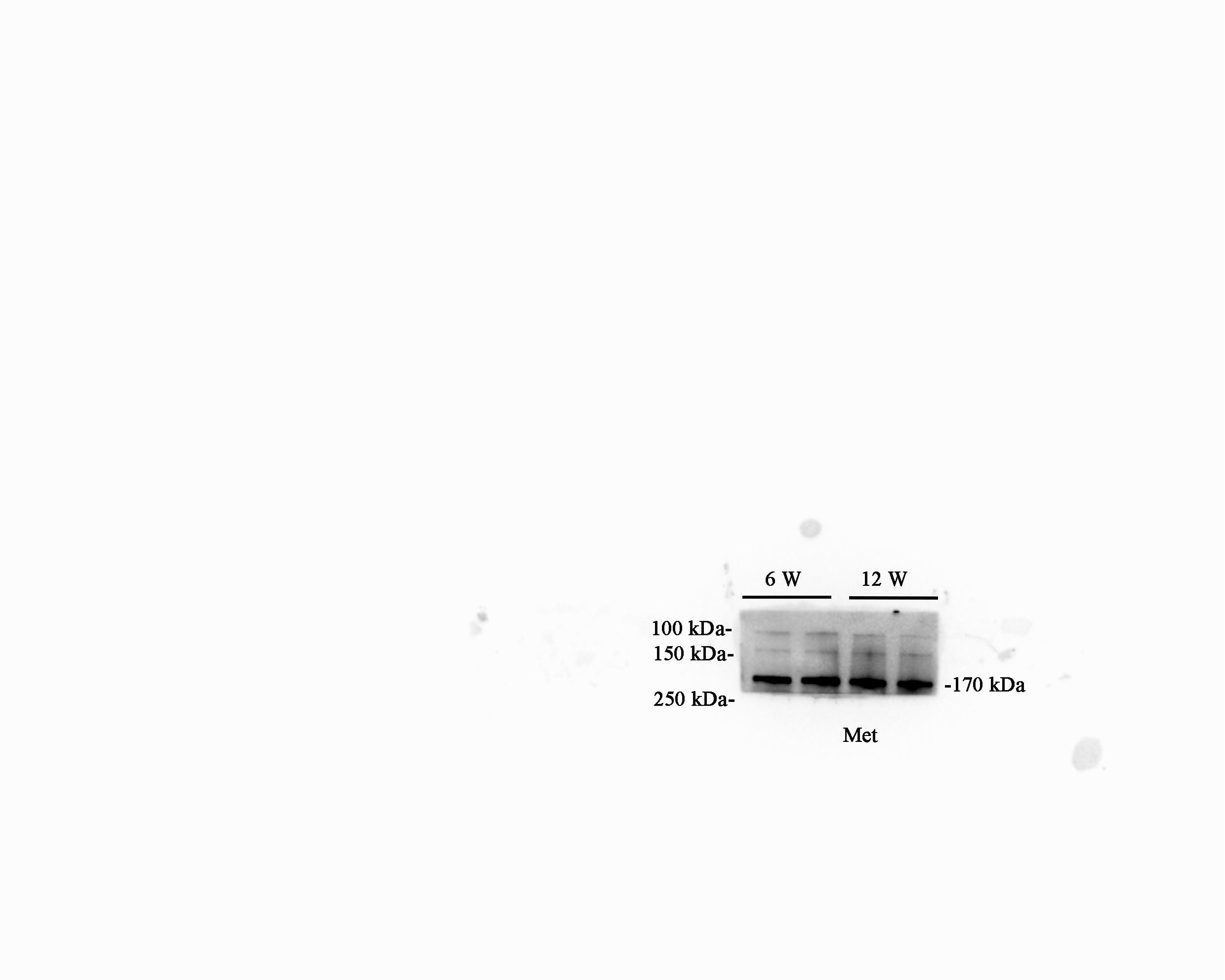


Figure 5E-Met


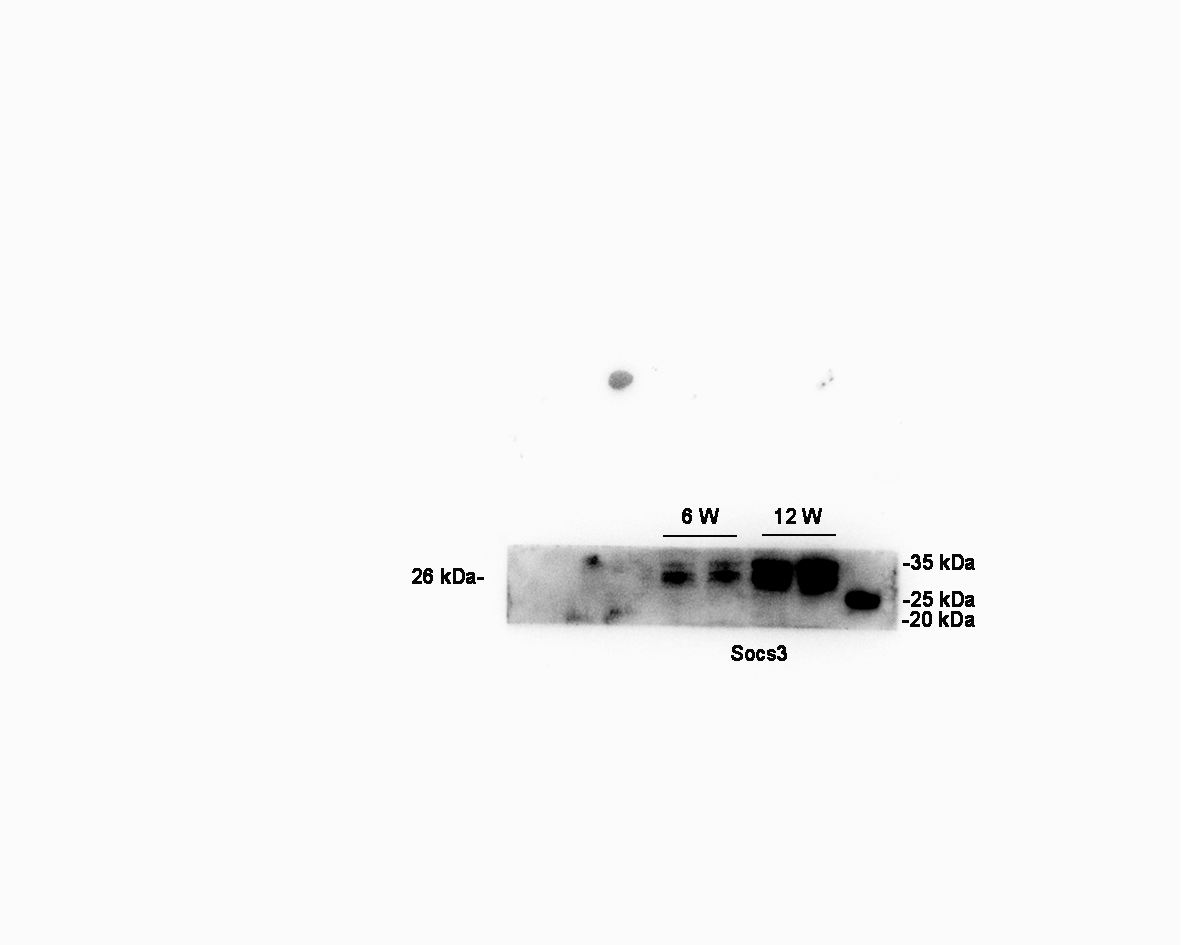


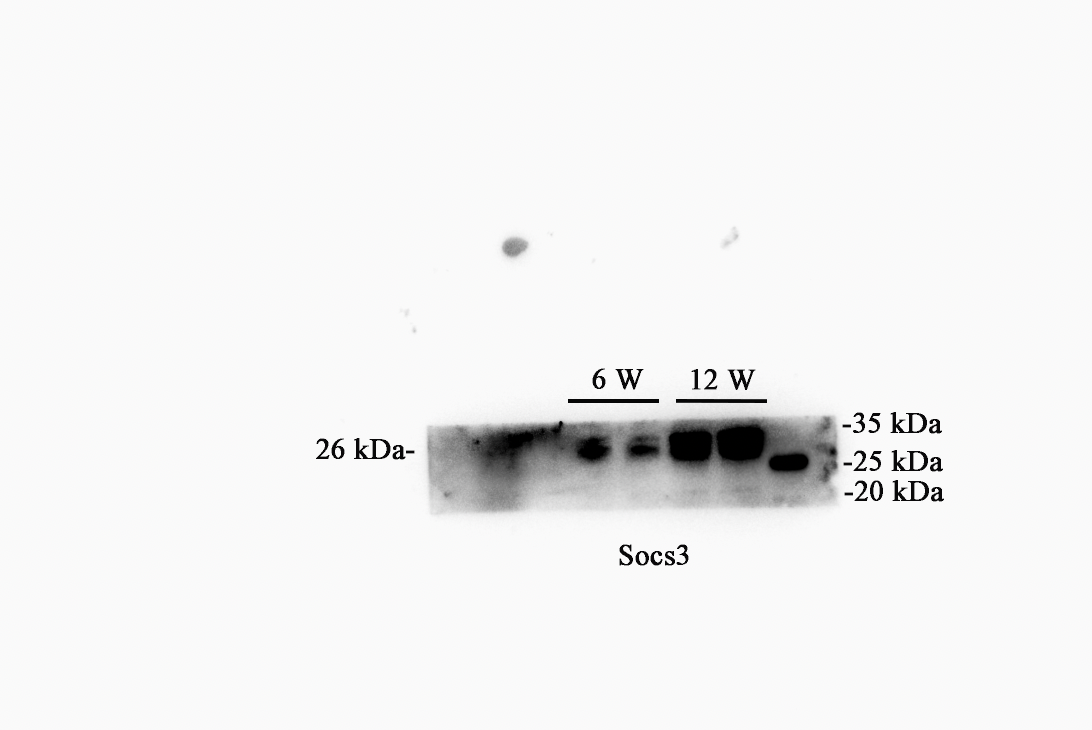


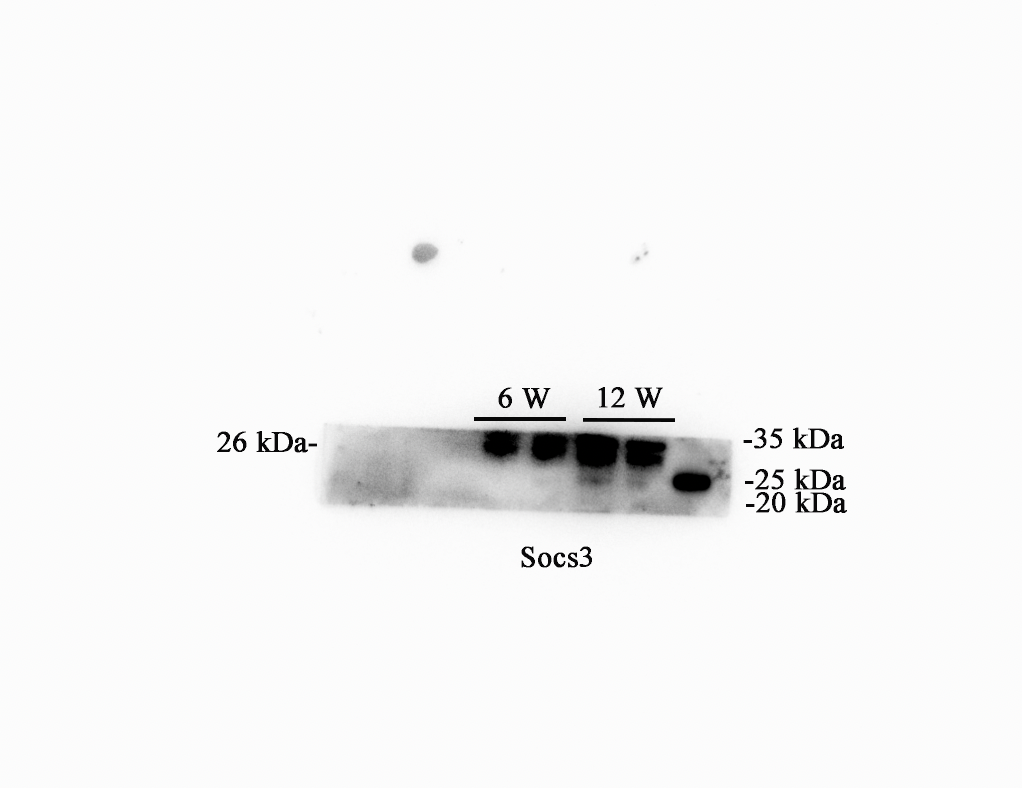


Figure 5E-Socs3


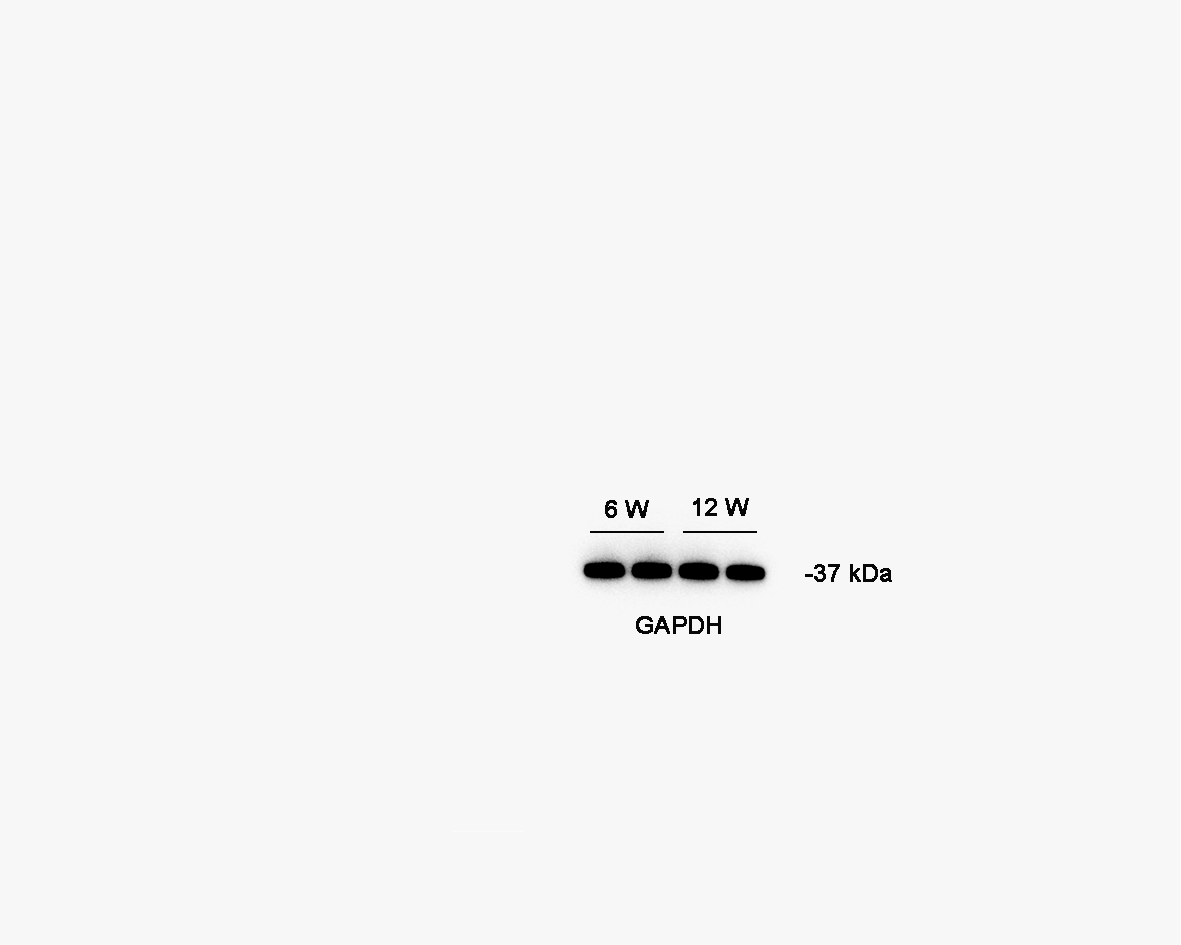


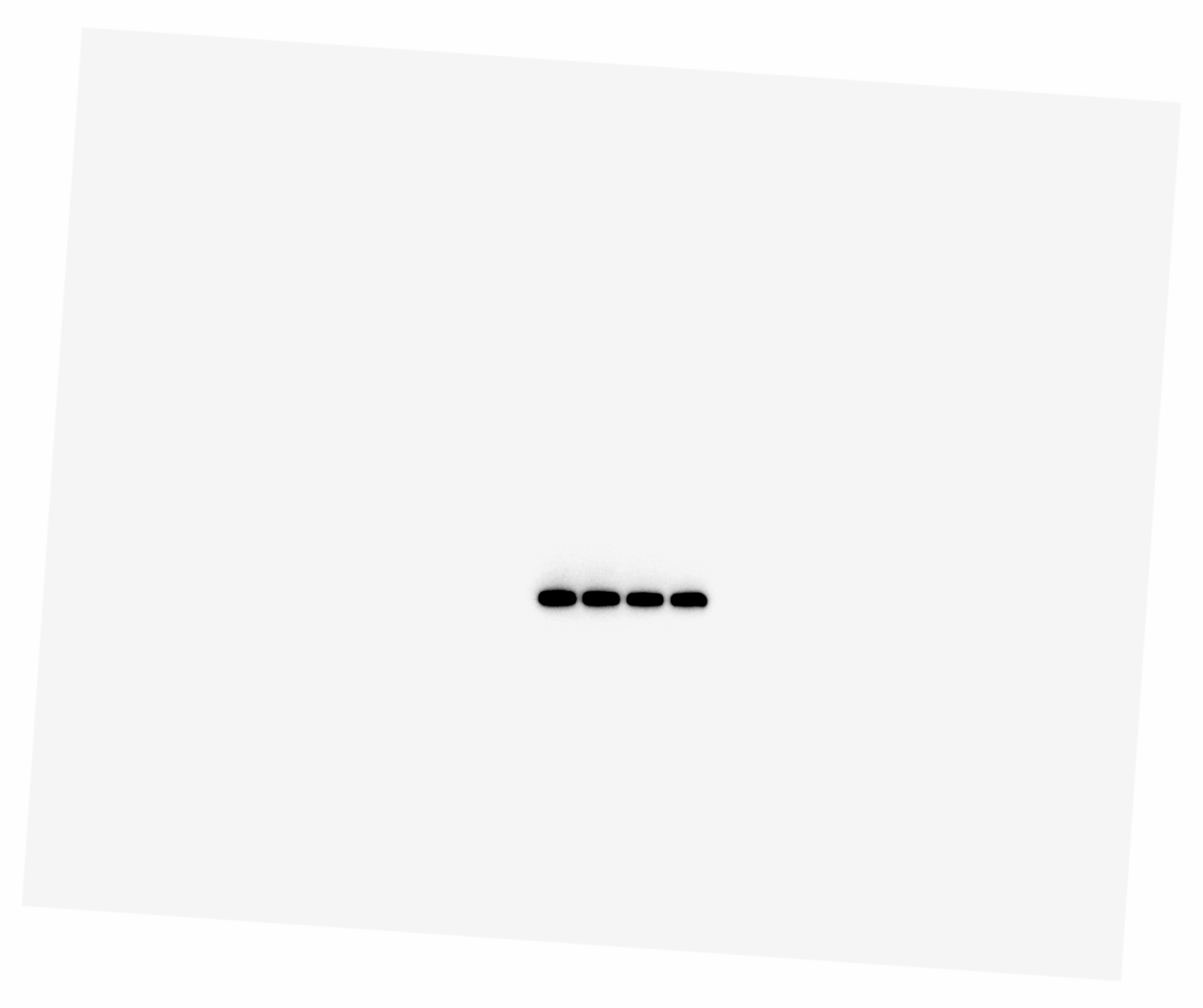


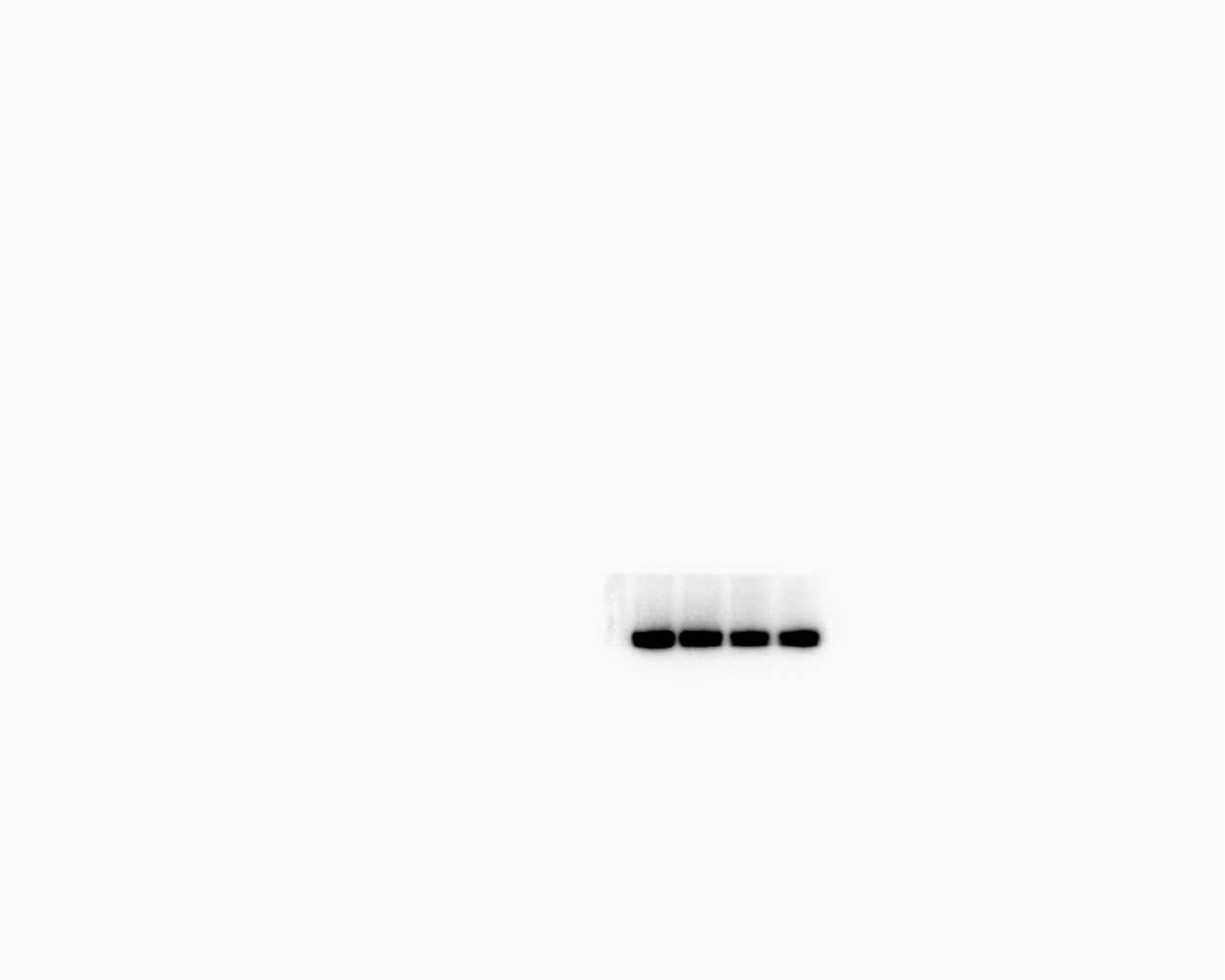


Figure 5E-GAPDH
