# Supplementary material for: Youthful systemic milieu in younger recipients alleviates acute kidney injury via attenuating apoptosis and oxidative stress in a rat kidney transplantation model
Source: PLoS One. 2025 Sep 23;20(9):e0331020. doi: 10.1371/journal.pone.0331020 (PMC12456805; doi:10.1371/journal.pone.0331020)
Supplement: Attachment — Submitted filename: Response to reviewers.docx [file pone.0331020.s007.docx]

**Responses to editor’s and reviewers’ comments**

-------------------------------------------------------------------------------------------------------
**- Journal Requirements:**

When submitting your revision, we need you to address these additional requirements.

1. Please ensure that your manuscript meets PLOS ONE's style requirements, including those for file naming.
[Response] Thank you so much. We have checked the whole manuscript carefully and revised these requirements as journal needed.

2.PLOS ONE now requires that authors provide the original uncropped and unadjusted images underlying all blot or gel results reported in a submission’s figures or Supporting Information files. This policy and the journal’s other requirements for blot/gel reporting and figure preparation are described in detail at https://journals.plos.org/plosone/s/figures#loc-blot-and-gel-reporting-requirements and https://journals.plos.org/plosone/s/figures#loc-preparing-figures-from-image-files. When you submit your revised manuscript, please ensure that your figures adhere fully to these guidelines and provide the original underlying images for all blot or gel data reported in your submission.

[Response] We fully understand the journal’s requirements on the original uncropped and unadjusted images. We provided the original uncropped and unadjusted western blot images (Fig. 3G, Fig. 5E) in supplementary materials, named “The original western blot images”.

3. Please note that PLOS ONE has specific guidelines on code sharing for submissions in which author-generated code underpins the findings in the manuscript. In these cases, we expect all author-generated code to be made available without restrictions upon publication of the work. Please review our guidelines at https://journals.plos.org/plosone/s/materials-and-software-sharing#loc-sharing-code and ensure that your code is shared in a way that follows best practice and facilitates reproducibility and reuse.

 [Response] All differently expressed genes (DEGs) were mapped to GO terms in the Gene Ontology database (<http://www.geneontology.org/>) and visualized using the online OmicStudio tools at: <https://www.omicstudio.cn/tool>, as stated on Methods, and no new author-generated code was available. In addition, all RNA-sequence data has been uploaded to the GEO database (accession number GSE226680), we also provided these statements in the manuscript.

4. To comply with PLOS ONE submissions requirements, in your Methods section, please provide additional information regarding the experiments involving animals and ensure you have included details on (1) methods of sacrifice, and (2) efforts to alleviate suffering.

 [Response] Thank you for you reminding. We have added the details of animal sacrifice and measures taken to alleviate suffering during the experiments in Methods 2.1/2.2, which marked with track changes in resubmitted manuscript. All animal procedures were approved by the Ethics Committee of Children’s Hospital of Chongqing Medical University (protocol code CHCMU-IACUC20220429006).

5. We note that the grant information you provided in the ‘Funding Information’ and ‘Financial Disclosure’ sections do not match.
When you resubmit, please ensure that you provide the correct grant numbers for the awards you received for your study in the ‘Funding Information’ section.

 [Response] Thank you very much. We checked the “Funding Information” in manuscript and the “Financial Disclosure” in the online submission system, the “Financial Disclosure” missed the Funding “Program for Youth Innovation in Future Medicine, Chongqing Medical University (W0201)”, we also included the updated statement in the cover letter.

6. Thank you for stating the following financial disclosure:
This research was funded by Chongqing Natural Science Foundation Innovation and Development Joint Fund Project (No. CSTB2024NSCQ-LZX0072), and The Joint Medical Research Project of Chongqing Science and Technology Bureau and Health Commission (No.2024MSXM002).
Please state what role the funders took in the study. If the funders had no role, please state: "The funders had no role in study design, data collection and analysis, decision to publish, or preparation of the manuscript."
If this statement is not correct you must amend it as needed.
Please include this amended Role of Funder statement in your cover letter; we will change the online submission form on your behalf.

[Response] We updated the Funding Information in cover letter, thank you.
7. Please review your reference list to ensure that it is complete and correct. If you have cited papers that have been retracted, please include the rationale for doing so in the manuscript text, or remove these references and replace them with relevant current references. Any changes to the reference list should be mentioned in the rebuttal letter that accompanies your revised manuscript. If you need to cite a retracted article, indicate the article’s retracted status in the References list and also include a citation and full reference for the retraction notice.

[Response] We have checked and updated the reference as journal requirements needed, these was no cited papers that have been retracted.

-------------------------------------------------------------------------------------------------------

**- Reviewer #1:**

My suggestion is to review the English and make some changing.
Here my suggestion:
1. In the Ethics Statement the Authors need to add the authorization number and the Authority that approved the study.
[Response] Thank you for your kind review, we have checked the whole manuscript carefully and revised these misspellings, grammatical errors, and wrong words. All animal procedures were approved by the Ethics Committee of Children’s Hospital of Chongqing Medical University (protocol code CHCMU-IACUC20220429006), we also stated this in the part of “Methods 2.1” and “Institutional review Board Statement” in the manuscript.
2. In the introduction part the authors introduced AKI without explaining the linkage with the paragraph about KTx and the linkage with CKD. And, why all the evaluation was performed at 24h after KTx, there is any previous study that report that the evaluation in 24h is sufficient for the study?
They could also specify the acronym OPTN/SRTR. In addition, the Authors speculate about graft and patient survival after KTx, but the results are still reported in OPTN/SRTR report.
[Response] Thank you for your careful review and constructive suggestions towards our manuscript. We already added the linkage between AKI and CKD/KTx in the introduction part as marked with track changes. Chronic kidney disease (CKD) is a worldwide health problem with about 690 million all-stage CKD patients and its prevalence is still increasing globally, kidney transplantation (KTx) is the optimal therapy for CKD, with higher level of body health, life quality, and social functions. However, acute kidney injury (AKI) is the most critical and early factor affecting the function and long-term survival of graft after transplantation, how to alleviate AKI after KTx is still a big problem for nephrologists and urologists. Thus, we would like to find out a novel strategy to ameliorate AKI and we conducted this study. Twenty-four hours after intervention is regarded as a reliable time point to study AKI according to literature reports[1-3], so we also tested the AKI levels 24 h after KTx, and it is sufficient for the study. In addition, we specified and added the acronym OPTN/SRTR in Introduction to make it more readable.

Reference:

1. Liu D, Lun L, Huang Q, Ning Y, Zhang Y, Wang L, et al. Youthful systemic milieu alleviates renal ischemia-reperfusion injury in elderly mice. Kidney Int. 2018;94(2):268-79. Epub 2018/06/25. doi: 10.1016/j.kint.2018.03.019. PubMed PMID: 29935950.

2. Yu C, Zhang J, Pei J, Luo J, Hong Y, Tian X, et al. IL-13 alleviates acute kidney injury and promotes regeneration via activating the JAK-STAT signaling pathway in a rat kidney transplantation model. Life Sci. 2024;341:122476. Epub 2024/02/08. doi: 10.1016/j.lfs.2024.122476. PubMed PMID: 38296190.

3. Duff S, Wettersten N, Horiuchi Y, van Veldhuisen DJ, Raturi S, Irwin R, et al. Absence of Kidney Tubular Injury in Patients With Acute Heart Failure With Acute Kidney Injury. Circ Heart Fail. 2024;17(11):e011751. Epub 2024/10/18. doi: 10.1161/circheartfailure.123.011751. PubMed PMID: 39421939; PubMed Central PMCID: PMCPMC11573103.
3. In the animals’ paragraph the Authors need to report the age of the rats even if they specify in the other paragraph. Why the authors performed a bilateral nephrectomy and replace only one kidney?
For the measurement of malondialdehyde levels (MDA), total superoxide dismutase activity (T-SOD), glutathione peroxidase activity (GSH-Px), and protein carbonyl content (PC) which type of commercial kits the Authors used?
For all the assays performed in paragraphs 2.8, 2.9 and 2.12 which typo of sample the Authors use for the assays, please specify.
[Response] We added the rats age in the animal’s paragraph as suggested. Patients with end-stage renal disease (ESRD) usually have both kidneys damaged, while kidney transplantation only requires one transplanted kidney for life-supporting in clinical practice, thus, we performed the bilateral nephrectomy and transplanted only one kidney in animal kidney transplantation models, as all researchers do. As methods 2.7 stated, kidney Malondialdehyde (MDA), total superoxide dismutase activity (T-SOD), glutathione peroxidase activity (GSH-Px) levels were tested by commercial kits purchased from Nanjing Jiancheng Biochemistry Co. (Nanjing, China); and protein carbonyl (PC) kits was purchased from Solarbio Science & Technology Co. (BC1275, Beijing, China). We also specified the sample types used in Methods 2.8, 2.9, and 2.12, Thanks a lot.
4. For both figure 3F and H I suggest a histogram, or percentage respect to total cells, with the number of positive cells, to better evaluate the results.

[Response] Thank you for your reminding, we took your advice and provided the histograms with the percentage of positive cells respect to total cells, to better evaluate the results.

-------------------------------------------------------------------------------------------------------

**- Reviewer #2:** 

In this paper the authors explore the effect of kidney transplantation from adult rats into young or adult recipients and evaluate how the recipient’s milieu influence kidney damage. While the results are interesting, I think that some points should be clearer:
1. While the difference in cytokines expression is interesting, I think that a qPCR or a western blot must be performed to confirm the expression’s difference; similar to what they did with SOC3 and MET.
[Response] Thank you for your kind review and positive attitudes towards our work. We performed the qPCR to confirm the expression of Akt2, ApoA1, and ApoE, which differently expressed by cytokine array. Also, we completed the corresponding part of Methods and Fig. 6. The expression of target protein was based on the formula 2^−ΔΔCt^, and the primers used were included in Supplementary Table 1, as:

Rat GAPDH, forward: 5'-CTGGAGAAACCTGCCAAGTATG-3', reverse: 5'-GGTGGAAGAATGGGAGTTGCT-3'.

Rat Akt2, forward: 5'-CCTTATGCTGGACAAAGATGGC-3', reverse: 5'- CCGTAGTCATTGTCCTCTAGCAC-3'.

Rat ApoA1, forward: 5'-AACAGCTAGGCCCAGTGACTCA-3', reverse: 5'- TCCTCGGCCACAACCTTTAGAT-3'.

Rat ApoE, forward: 5'-TGACGGTACTGATGGAGGACACT-3', reverse: 5'- CCAGCATGGTGTTTACCTCGTT-3'.
2. The authors assume the age of 6 weeks as young recipients and 12 weeks as old one, is there anything in literature that can confirm this assumption?
[Response] As rats reach sexual maturity at 6–8 weeks and physical maturity at 8–10 weeks, we chose 6- and 12-week-old rats as recipients to mimic pediatric/young KTx and adult KTx, respectively. In order to study the younger youthful systemic milieu in young recipients on AKI after kidney transplantation, we conducted the animal transplantation model with different age[1]: 12-week SD rat donor kidneys were heterotopically transplanted into 6-week Wistar rats (young recipients, Y group) and 12-week Wistar rats (adult recipients, A group). We also explained the choice of recipient age in Discussion part: in literature reports, young (<8 weeks) and very old (19–26 weeks) rodents have been routinely used for parabiotic connection to study the influence of youthful systemic milieu on old tissues[2,3], however, very old organs are out of consideration for clinical solid organ transplantation, so we did not select the very old rats as recipients, and instead, we chose 6- and 12-week-old rats as recipients to mimic pediatric and adult KTx, respectively, to identify whether the younger internal environment would ameliorate allograft injury. Interestingly, the choice of these age has certain innovative significance in the aspect of youthful systemic milieu.

Reference:

1. Yu C, Zhang J, Pei J, Luo J, Hong Y, Tian X, et al. IL-13 alleviates acute kidney injury and promotes regeneration via activating the JAK-STAT signaling pathway in a rat kidney transplantation model. Life Sci. 2024;341:122476. Epub 2024/02/08. doi: 10.1016/j.lfs.2024.122476. PubMed PMID: 38296190.

2. Baht GS, Silkstone D, Vi L, Nadesan P, Amani Y, Whetstone H, et al. Exposure to a youthful circulaton rejuvenates bone repair through modulation of β-catenin. Nat Commun. 2015;6:7131. Epub 2015/05/20. doi: 10.1038/ncomms8131. PubMed PMID: 25988592; PubMed Central PMCID: PMCPMC4479006.

3. Liu D, Lun L, Huang Q, Ning Y, Zhang Y, Wang L, et al. Youthful systemic milieu alleviates renal ischemia-reperfusion injury in elderly mice. Kidney Int. 2018;94(2):268-79. Epub 2018/06/25. doi: 10.1016/j.kint.2018.03.019. PubMed PMID: 29935950.
3. The kit information used to perform some experiments should be clarified, such as the ones for the measurements of malondialdehyde levels (MDA), total superoxide dismutase activity (T-SOD), glutathione peroxidase activity (GSH-Px), and carbonyl protein content (PC). In general, I suggest better specifying the method used instead of citing previous publication.
[Response] Thank you for your suggestions, we provided the commercial kit information in Methods 2.7: kidney Malondialdehyde (MDA), total superoxide dismutase activity (T-SOD), glutathione peroxidase activity (GSH-Px) levels were tested by commercial kits purchased from Nanjing Jiancheng Biochemistry Co. (Nanjing, China); and protein carbonyl (PC) kit was purchased from Solarbio Science & Technology Co. (BC1275, Beijing, China). In addition, we have specified the methods, which marked with track changes in resubmitted manuscript.
4. The ethic statement should be filled since this work includes the use of vertebrate animals.
[Response] We have included and filled the ethic statement, all animal procedures were approved by the Ethics Committee of Children’s Hospital of Chongqing Medical University (protocol code CHCMU-IACUC20220429006), we also stated this in the part of “Methods 2.1” and “Institutional review Board Statement” in the manuscript.
5. I think that there is some confusion in image citations within the text, they cite figure 5-6 with data that are in figure 4-5. So, I suggest thoroughly performing a proof reading to be sure that there are not more typos and figures wrongly cited that make it harder to follow the work.

[Response] Thank you for your careful review, we have carefully checked the order of Figure citations, to ensure that all the figures and tables were cited in the correct position and in sequence.

-------------------------------------------------------------------------------------------------------

**- Reviewer #3:**

The Authors evaluate “renal replacement dialysis therapy and kidney transplantation (KTx) are the only choices for life-supporting for ESRD patients.”
Why the Authors never evaluate the possibility to describe, and evaluate the possibility of islet transplantation and describe in the introduction or in the discussion the possible advantages or disadvantages instead a whole kidney transplantation?
The Authors report in the conclusion “Cytokine therapy may be a potentially useful therapeutic approach in kidney transplanted recipients to minimizing the injury of oxidative stress and apoptosis and to maximize the protection of kidney function and repair after injury.”
Here some paper to evaluate that evaluate the advantages of islet transplantation instead to perform a whole kidney transplantation. The Authors could discuss the advantages of islet transplantation in combination with the cytokines they proposed in the paper.
• Islet transplantation is associated with an improvement of cardiovascular function in type 1 diabetic kidney transplant patients (P. Fiorina et al. Diabetes Care 2005)
• Long-term beneficial effect of islet transplantation on diabetic macro-/microangiopathy in type 1 diabetic kidney-transplanted patients (P. Fiorina Diabetes Care 2003)

[Response] Thank you for your careful review and constructive suggestions, islet transplantation was not given sufficient attention among the kidney transplantation that I previously concerned about. The combination of cytokines and islet transplantation may provide more promising prognostic results in patients with ESRD underwent kidney transplantation. I do believe this aspect you taught me would affect my subsequent work greatly. These 2 papers were well designed and conducted, which proposed the possible benefits of islet transplantation in improving cardiovascular function and long-term survival in type 1 diabetic kidney-transplanted patients, we added this point in Discussion part and cited these papers. Thank you again!

-------------------------------------------------------------------------------------------------------

We tried our best to improve the manuscript and made some modification in the manuscript. The modification will not influence the content and framework of the paper.
 We appreciate for your warm work earnestly, and hope that the correction will meet with approval.

Once again, thank you all for your comments and suggestions.
